# Tropomyosin and Actin Identified as Major Allergens of the Carpet Clam (*Paphia textile*) and the Effect of Cooking on Their Allergenicity

**DOI:** 10.1155/2015/254152

**Published:** 2015-08-27

**Authors:** Zailatul Hani Mohamad Yadzir, Rosmilah Misnan, Faizal Bakhtiar, Noormalin Abdullah, Shahnaz Murad

**Affiliations:** ^1^Allergy and Immunology Research Centre, Institute for Medical Research, Jalan Pahang, 50588 Kuala Lumpur, Malaysia; ^2^Department of Biology, Faculty of Science and Mathematics, Universiti Pendidikan Sultan Idris, 35900 Tanjong Malim, Perak, Malaysia

## Abstract

*Objectives*. To identify the major allergenic proteins of clam (*Paphia textile*) and to investigate the effect of different cooking methods on the allergenicity of these identified proteins. *Methods*. Clam protein extracts were separated by denaturing polyacrylamide gel electrophoresis. IgE reactive proteins were then analyzed by immunoblotting with sera from patients with positive skin prick tests (SPT) to the raw clam extract. Mass spectrometry was used to identify the major allergenic proteins of this clam. *Results*. Raw extract showed 12 protein bands (18–150 kDa). In contrast, fewer protein bands were seen in the boiled extract; those ranging from 40 to 150 kDa were denatured. The protein profiles were similarly altered by frying or roasting. The immunoblots of raw and boiled extracts yielded 10 and 2 IgE-binding proteins, respectively. The fried and roasted extracts showed only a single IgE-binding protein at 37 kDa. Mass spectrometry analysis of the 37 and 42 kDa major allergens indicated that these spots were tropomyosin and actin, respectively. *Conclusion*. The two major allergens of *Paphia textile* were identified as the thermostable tropomyosin and a new thermolabile allergen actin.

## 1. Introduction

Clams are an important variety of shellfish and perhaps the most versatile seafood in the world [1]. Clams are high in protein and the nutritive value of several species of clams has been estimated [2, 3]. Clam meat has been recommended in several dietary regimes for its high protein content, low caloric value, low fat/cholesterol profile and lower proportion of saturated fat, the presence of unsaturated lipids, significant amount of omega-3 fatty acids, dietary essential amino acids, vitamin B_12_, and several important minerals such as iron, zinc, and copper [1].

*Paphia textile*, locally known as* lala* (carpet clam), is one of the more popular edible shellfish in Malaysia. However, shellfish including the carpet clam appear to be a significant cause of allergy among local patients with asthma and allergic rhinitis [4]. Upon consumption, inhalation, or contact with clams, sensitive individuals may experience multiple symptoms [5]. The frequently observed symptoms include angioedema, vomiting, urticaria, allergic rhinitis, diarrhea, skin rash, swelling of the tongue or throat, and asthma [5–8].

To date, there are only a few reports on the identification of clam allergens. Tropomyosin is the only allergen that has been identified in different clam species. Emoto et al. [9] reported the 37 kDa major allergen in the surf clam* Pseudocardium sachalinensis*, razor clam* Solen strictus*, and horse clam* Tresus keenae* as tropomyosins. Tropomyosin embraces a group of highly conserved proteins with a molecular weight between 34 and 38 kDa. It is present in muscle and nonmuscle cells and plays a central role in muscle contraction [10–12]. It has been well identified as an allergen for various types of shellfish [9, 13–18].

Different processing methods play an important role in modifying the allergenicity [18–20]. These processes decrease, enhance, or sometimes have no effect on the allergenicity [20–22]. Most clams are cooked either by boiling, roasting, or frying, all of which can be considered a form of thermal treatment. Given the pivotal role of the process of cooking in clam allergenicity, this study investigated the effect of different cooking methods on the allergenicity of clam and aimed at identifying the major allergenic proteins of the clam (*Paphia textile*).

## 2. Materials and Methods

### 2.1. Clam Protein Extraction

The clam shell was split open and the inner muscle tissue was removed and used for extraction. About 20 g of the muscle mass was homogenized in 200 mL of 0.1 M phosphate buffered saline (PBS), pH 7.2, for 10 minutes using a Waring blender. This homogenate was then agitated overnight at 4°C followed by centrifugation at 4,500 and 14,000 rpm for 30 and 15 minutes, respectively. The clear supernatant was then recovered and sterilized by passage through a 0.22 *μ*m syringe filter, frozen, and lyophilized. The lyophilized extracts were stored at −20°C until used. The boiled clam extract was prepared by boiling muscle tissue with 0.1 M PBS (pH 7.2) for 10 minutes at 100°C before homogenization in a Waring blender. The fried clam extract was obtained by frying the muscle tissue in vegetable oil for 10 minutes at 120°C and subsequently dried on filter paper to remove the oil. The fried clam muscle was then homogenized in 0.1 M PBS (pH 7.2) and extracted as above. The roasted clam extract was obtained by roasting at 180°C for 10 minutes followed by homogenization using the same protocol as above.

Protein concentration of the extracts was determined using the total protein kit (Sigma-Aldrich, USA) according to the manufacturer's instructions. Bovine serum albumin (BSA) was used as the protein standard. 0.5 mL of the protein standard was made up to 50 mL of 0.85% sodium chloride solution. 5 different concentrations of diluted protein standard were then prepared: 0, 250, 500, 750, and 1,000 *μ*g/mL. 2.2 mL Biuret reagent was added to each tube and mixed well. This mixture was incubated at room temperature for 10 minutes. Then, 100 *μ*L of Folin and Ciocalteau's phenol reagent was added followed by incubation for 30 minutes in the absence of light. The colour intensity of the protein standards was measured using spectrophotometer at 650 nm wavelength and a graph of absorbance against protein concentration was plotted to get a standard calibration curve. For preparation of sample, lyophilized allergen extract was reconstituted with 500 *μ*L of PBS followed by centrifugation at 14,000 rpm for 5 minutes. 20 *μ*L of the supernatant was collected and then diluted with 180 *μ*L of 0.85% sodium chloride solution (ratio 1 : 9) and processed in the same manner as described above. The protein content of samples was then estimated by comparing their measurements with the standard calibration curve. All protein standards and samples were carried out in triplicate.

### 2.2. Experimental Sera

Sera of 21 patients with a history of clam allergy and a positive skin prick test (SPT) to raw clam extract were used in this study. The sera were obtained from patients referred to the Allergy Clinic, Kuala Lumpur Hospital, while control sera were obtained from nonallergic subjects. The sera were stored at −80°C until used. This study was approved by the Medical Research and Ethics Committee (MREC), Ministry of Health, Malaysia.

### 2.3. Sodium Dodecyl Sulphate-Polyacrylamide Gel Electrophoresis (SDS-PAGE) and Immunoblotting

SDS-PAGE was carried out with a 12% polyacrylamide separating gel and a stacking gel of 5%. Electrophoresis was done on a Mini-PROTEAN 3 Apparatus (Bio-Rad, USA) at 120 mA for 45 minutes. Each sample was dissolved in Laemmli sample buffer (Bio-Rad) in the presence of 5% 2-mercaptoethanol, heated at 97°C for 4 minutes, and subjected to electrophoresis. Precision plus protein standards (Bio-Rad) were run as reference along with samples. After running, the gel was stained with Coomassie Brilliant Blue R-250 (Bio-Rad). Protein mass was estimated by comparing the clam protein bands with the molecular weight markers using Imaging Densitometer GS800 and Quantity One software (Bio-Rad).

Following SDS-PAGE, the separated proteins were electrotransferred from the gel to a 0.45 *μ*m nitrocellulose membrane using the Mini Trans-Blot System (Bio-Rad) at 100 V for 70 minutes. The membrane was then stained with Ponceau S dye (Sigma-Aldrich, USA) to verify transfer of the separated proteins. Strips of 3 mm width were cut from the membrane and washed with Tris-buffered saline (TBS) containing 0.05% Tween 20 (TTBS) and nonspecific binding was blocked with 10% nonfat milk in TBS. The strips were incubated with the individual patients' sera in blocking buffer overnight at 4°C. IgE-binding proteins were then detected using biotinylated goat antihuman IgE antibody (Kirkegaard and Perry Laboratories, UK) followed by incubation with streptavidin-conjugated alkaline phosphatase (Bio-Rad) for 30 minutes at room temperature. Finally, the alkaline phosphatase conjugate substrate (Bio-Rad) was used for color development. Serum from a nonallergic subject was used as a negative control, while a strip without a serum sample was used as blank.

### 2.4. Two-Dimensional Gel Elctrophoresis (2-DE) and Immunoblotting

The raw clam extract was suspended in rehydration buffer containing 8 M urea, 50 mM DTT, 4% chaps, 0.2% carrier ampholyte, pH 3–10, and 0.0002% bromophenol blue. 50 *μ*g protein sample was then applied to an immobilized nonlinear pH 3–10 gradient strip of 7 cm length (Bio-Rad, USA) for rehydration overnight (12–14 hours). Isoelectric focusing was run using the Protean IEF Cell Apparatus (Bio-Rad) with the following voltage/time gradient: 100 V for 1 minute, 250 V for 30 minutes, 4 000 V for 2 hours, and 4 000 V for 10 000 V-hr (Vhour). Before transferring the immobilized pH gradient (IPG) strip onto the second dimension, the strip was equilibrated sequentially for 10 minutes in a buffer containing 65 mM dithiothreitol and then 135 mM iodoacetamide in 125 mM Tris-HCl, pH 6.8, 6 M urea, 2% SDS, 30% glycerol, and 0.01% bromophenol blue. After equilibration, the strips were placed onto 12% SDS-PAGE separating gels with 5% of stacking gels and sealed in place using Ready Prep Overlay Agarose (Bio-Rad) for second dimension. The resultant gels were either stained for protein with Coomassie Brilliant Blue R-250 (Bio-Rad), scanned using Imaging Densitometer GS800 and analysed using PDQuest software (Bio-Rad), or subjected to protein transfer and immunoblotting as described above for SDS-PAGE and immunoblotting.

### 2.5. Mass Spectrometry Analysis

The Coomassie-stained protein spots corresponding to those recognized by the above sera were manually excised and transferred to microcentrifuge tubes. These protein spots were analyzed using mass spectrometry analysis by First Base Laboratories Sdn Bhd, Malaysia. Protein samples were trypsin digested and the peptides were extracted according to standard techniques. These peptides were analyzed by matrix-assisted laser desorption-ionization time of flight (MALDI-TOF) mass spectrometer using a 4800 Proteomics Analyzer. Spectra were analyzed to identify the protein of interest using Mascot sequence matching software (Matrix Science) with Ludwig NR Database and taxonomy set to other metazoa.

## 3. Results

### 3.1. Comparison of Protein Fractions in Raw, Boiled, Fried, and Roasted Clam Extracts by SDS-PAGE

**Figure 1:**
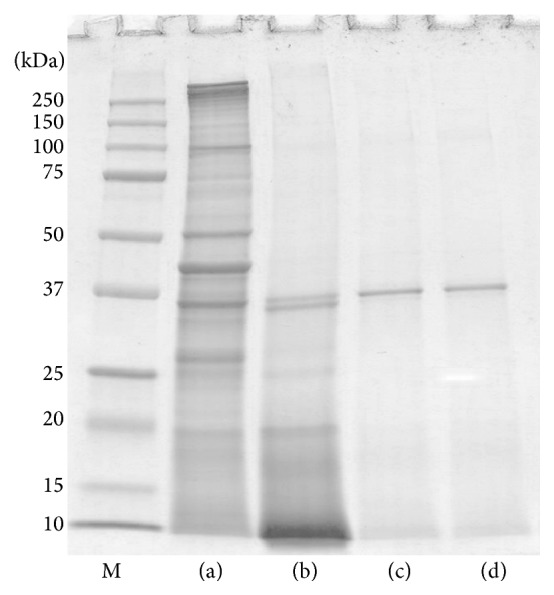
SDS-PAGE profiles of raw (a), boiled (b), fried (c), and roasted (d) extracts of* Paphia textile*.* Lane M*, molecular mass markers.

SDS-PAGE of the raw clam extract demonstrated 12 protein bands in the 18 to 150 kDa range. The cooked clam extracts, on the other hand, showed fewer protein bands. While the protein bands in the 40 to 150 kDa range of the raw clam were not found in the boiled extract, most of the protein bands were missing from SDS-PAGE of the fried and roasted extracts. However, 37 kDa was conserved in all the extracts regardless of the cooking process employed (Figure 1).

### 3.2. Comparison of IgE-Binding to Proteins from Raw and Cooked Clam Extracts

**Figure 2:**
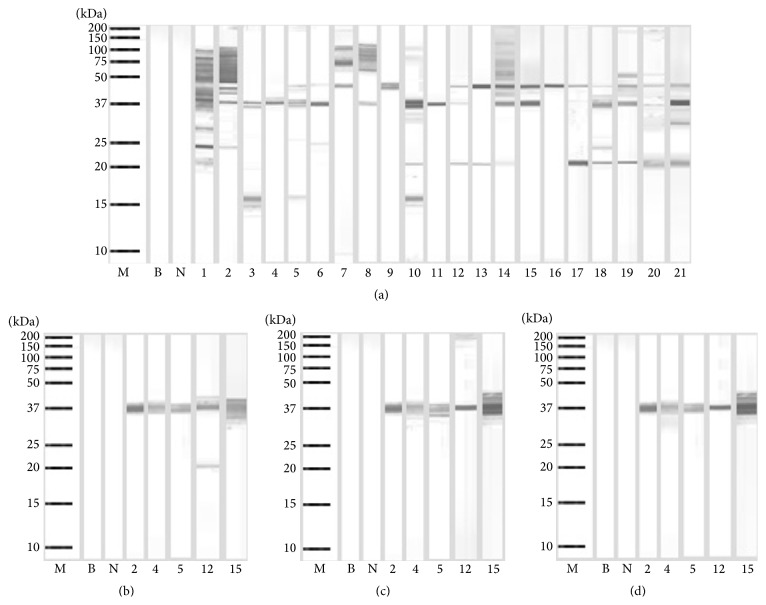
Immunoblotting of raw (a), boiled (b), fried, (c) and roasted (d) extracts of* Paphia textile*.* Lane M*, molecular mass markers;* lanes 1–21*, immunoblots showing binding of IgE from different serum samples;* lane N*, immunoblot using serum from a nonallergic individual; and* lane B*, blank.

The immunoblot of the raw extract displayed 10 IgE-binding proteins between 16 to 100 kDa (Figure 2(a)). The 37 and 42 kDa proteins exhibited the highest frequency of IgE-binding, 81%, and thus were identified as the major allergens of this clam. While the 20 kDa protein was detected by sera from 48% of the patients; the 50, 65, 75, and 100 kDa proteins bound IgE from 24 to 29% of the sera from these patients. The 16, 25, and 29 kDa IgE-binding proteins were recognized by 14–19% of the patients.

The allergenicity of the boiled, fried, and roasted clam extracts was further studied using 5 allergic patients' sera, as shown in Figures 2(b), 2(c), and 2(d). These patients' sera were selected because their IgE binds to the proteins of the raw extract seen in the immunoblot above. All five patients' sera held 37 kDa binding IgE and most sera showed staining of this protein band, indicating that this is a major allergen in this clam. In addition to IgE binding the 37 kDa protein, one serum showed IgE binding to the 20 kDa protein found in the boiled clam extract. No IgE-binding was observed in the negative control serum.

### 3.3. 2-DE Profile and IgE-Binding Spot Analysis

**Figure 3:**
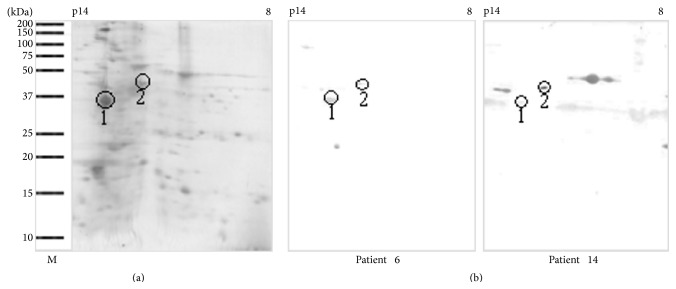
Two-dimensional electrophoresis and immunoblot analysis of* Paphia textile*. (a) Coomassie blue stained blot. (b) Immunoblot with individual patients' sera. Spot number 1 and spot number 2 were sent for mass spectrometry analysis.* Lane M*, molecular mass markers.

Coomassie blue staining of the separated protein components showed ~50 distinct spots, with molecular weights between 18 to 150 kDa and isoelectric points (pI) ranging from 3.0 to 10.0 (Figure 3(a)), whereas immunoblotting detected less than 10 IgE-binding proteins (Figure 3(b)). This study focused on further identification of the allergenic spots of 37 and 42 kDa proteins, the major allergens of clam. Thus, spot number 1 (37 kDa/pI 4.7) and spot number 2 (42 kDa/pI 5.5) were selected for mass spectrometry analysis. No IgE-binding spots were detected by immunoblotting using a control serum from a nonallergic subject (result not shown).

### 3.4. Allergen Identification

Table 1 summarizes the mass spectrometry analysis of the spots. Sequence comparisons of a 37 kDa spot (spot 1) and a 42 kDa spot (spot 2) with known protein sequences in databases have identified these spots as tropomyosin and actin, respectively.

## 4. Discussion

Generally, clams are cooked prior to serving for palatability and safety from microbial contamination. Despite the advantages of cooking, which can be considered a form of thermal treatment, significant changes occur in the proteins through denaturation, that is, loss in tertiary and/or secondary interactions, formation of new intramolecular or intermolecular bonds, aggregation, and/or rearrangements of disulfide bonds as well as other conformational modifications, which can ultimately lead to changes in apparent allergenicity [19, 23]. Our study demonstrated that the process of cooking by boiling, frying, or roasting produces changes in the SDS-PAGE protein profile of clam allergens. Compared to the raw extract, boiling causes a remarkable reduction in the number of protein bands due to denaturation of proteins found within 40 to 150 kDa range. Both frying and roasting showed a similar protein profile, with most of the bands clearly eliminated compared with those of the raw and boiled extracts. The loss of the proteins in boiled, fried, and roasted extracts may be related to the effects of heat on protein structure, since heat may disrupt secondary and tertiary protein structures and lead to random-coiled aggregation and insolubility [14, 24]. In contrast, a prominent heat-resistant 37 kDa protein was detected in SDS-PAGE even after boiling, frying, or roasting.

Allergens are often characterized by having the following traits: the ability to sensitize a genetically predisposed individual by triggering the production of IgE antibodies, the ability to bind those particular IgE antibodies, and the ability to cause an allergic reaction following IgE-binding [25, 26]. Major allergens are defined on the basis of the frequency of recognition by serum IgE antibodies; that is, a frequency of greater than 50% justifies a designation as a major allergen [27]. In this study, a protein with molecular mass of 37 kDa was detected in 81% of the sera tested; thus, this 37 kDa protein was identified as one of the major allergens of this clam. This 37 kDa protein is heat stable because it retained its IgE reactivity in immunoblots of extracts from cooked clam. A majority of the sera had markedly increased IgE-binding intensity to the 37 kDa protein in the cooked clam extract suggesting that some new epitopes might have been exposed for IgE-binding [19–22].

In this study, we also detected another major allergen at 42 kDa. Interestingly, this 42 kDa major allergen and several additional minor allergens were detected only in the raw clam extract. This suggests that all these allergens were heat-sensitive proteins. The process of cooking may have altered the allergen extracts as a result of changes in protein conformation by masking the allergenic epitopes. Therefore, allergen recognition was reduced and so was allergenicity [20–22].

So far, tropomyosin was the only major allergen identified in different clam species [9]. Tropomyosin with a molecular weight around 34 to 38 kDa comprises a group of highly conserved actin-binding proteins present in muscle and nonmuscle cells and plays a central role in muscle contraction [10–12]. Similarly, our study has also identified the 37 kDa protein as tropomyosin by mass spectrometry analysis.

In this study, we have also identified for the first time that actin is the second major allergen of clam. It is a heat-sensitive protein with a molecular weight of 42 kDa. Thus far, only Abdel Rahman et al. [28] have identified *α*-actin as an allergenic protein in the snow crab* Chionoecetes opilio*. Actin is an important contractile protein in eukaryotic cells. It is one of the two major components involved in the contraction of muscle cells. In nonmuscle cells, it is the major part of the cytoskeleton involved in many processes such as cell motility, endocytosis, exocytosis, phagocytosis, organelle movement, material transportation, and signal transduction [29, 30]. Xiong and Blanchard [31] have demonstrated using SDS-PAGE that myosin and actin are not present in the supernatant when a suspension of myofibrils is heated at 65°C. Moreover, it was reported that actin denaturation occurred between 80 and 83°C when investigated using scanning calorimetry [32].

## 5. Conclusion

Our study has identified two major allergens with different properties which play an important role in local patients allergic to clam. One was a heat-resistant 37 kDa protein which corresponds to tropomyosin, and the other was a heat-sensitive 42 kDa protein, identified as actin.

## Figures and Tables

**Table 1:** Identities of major protein spots of carpet clam (*Paphia textile*) identified by mass spectrometry analysis.

Spot number	MW (kDa) and pI of matched proteins: observed/predicted	Protein identification	Organism	Accession number	Residue numbers of matched regions	Coverage of protein sequence
1	37/32.5 kDa, 4.7/4.6	Tropomyosin	*Balanus rostratus*	A2V716	34–46, 153–178, 205–238	25%
2	42/41.7 kDa, 5.5/5.3	Actin	*Haliotis discus discus*	B6RB19	21–30, 240–255	6%
